# A Tactful Prompt: The Time is Right for *Critical Behavioral Studies*

**DOI:** 10.1007/s40614-025-00472-2

**Published:** 2025-08-18

**Authors:** David Jackson-Perry, Elsa Suckle, Nick Chown, Jonathan Tarbox

**Affiliations:** 1 Queen’s University, Belfast, UK; 2 Bath Spa University, Bath, UK; 3 Independent Scholar, Birmingham, UK; 4 University of Southern California and FirstSteps for Kids, Los Angeles, CA USA

**Keywords:** Applied behavior analysis, Positive behavior support, Critical Autism Studies, Neurodiversity, Critical behavioral studies

## Abstract

Feelings have long run high between many autistic advocates and behavior analysts. The former often experience and perceive ABA as harmful and traumatic in its methods, and prejudicial and stigmatizing in its objectives, with some of the latter retorting that criticisms reflect misunderstandings of the science rather than areas of true concern. The result? A deep and contentious conceptual divide, leaving little room for dialogue or progress. Recent months, though, have seen a tentative shift. Alongside recognition that behavioral interventions are so deeply entrenched that they are here to stay, some critical autism scholars are gingerly initiating public conversations with behavioral practitioners in a spirit of taking a pragmatic approach to meaningful reform. Further, a new generation of behavior analysts—including some autistic practitioners—is emerging, recognizing problems in their field, and considering how to address them. Interest in such developments is spreading and signals an opportunity for behavior analysts to follow other academic and advocate communities that recognize the importance of interdisciplinarity and critical self-reflection to evolve as a field. We—an interdisciplinary team of critical autism, neurodiversity, and behavior analysis scholars—feel that formalizing a broad field for scholars and practitioners sharing these ambitions holds potential. This field—let’s call it *Critical Behavioral Studies*—would favor profound social, cultural, and historical understanding, a commitment to extend the scope of training to better contextualize practice in relation to the group served, and the self-examination that would bring meaningful change to the field.

There is concern among some autistic laypeople, advocates, and academics that behavioral interventions such as applied behavior analysis (ABA) and positive behavior support (PBS) are inherently and irrecoverably flawed and harmful. Critiques are not limited to implementation and methods perceived as cruel or abusive. They extend to what some of us consider to be the problematic history of ABA leading to a perception that any behavioral practice constitutes “fruit of the poisoned tree”; inadequate training and a lack of autism-specific or broader psychology training within many ABA training programs, with the resultant total ignorance of some practitioners about what it means to live as an autistic person; and the reality that ABA is a multibillion-dollar industry with the ethical concerns that this and all the preceding points here entail (Shkedy et al., 2021). It is encouraging that some behavior analysts, including certain contributors to the special issue in which this invited article appears, share some of these concerns.

Public and academic debates between autistic and behavior-analytic communities had not previously brought any kind of resolution, or even understanding, with tit-for-tat accusations and denials leading to dead-ends rather than dialogue. But this impasse appears to be shifting. Autistic ABA practitioners have emerged, such as Johnson (2025), who proposes that ABA-based practice serving the autistic community center autistic perspectives in defining its ethical guidelines, intervention goals, and systemic policies. A new generation of autistic scholars and behavior analysts are engaging in critical exchange, recognizing that where behavioral interventions are so deeply and systematically entrenched they are not going anywhere anytime soon. Recent publications authored by neurodiverse teams propose pathways for further reflecting on and solidly integrating neurodiversity-affirming principles into behavioral interventions, such as commitments to autonomy, assent-based practice, and the rejection of goals rooted in neurotypical conformity (Allen et al., 2024; Bambara et al., 2025; Kilgallon et al., 2025; Mathur et al., 2024; Veneziano & Shea, 2023). Others are taking the risk of putting apparently opposing stakeholders into dialogue (Suckle et al., 2025) or calling for the field to embrace cultural humility and social justice, echoing wider shifts in disability research and advocacy (Mathur & Rodriguez, 2022). These efforts hold considerable potential: doors that were previously slammed shut seem to be tentatively ajar.

The current special issue on challenges to ABA potentially pushes the door open a little further, representing an ideal opportunity to invite and put into dialogue a truly representative range of viewpoints. It is perhaps somewhat unfortunate that such limited inclusion of autistic voices was achieved both in terms of article authorship and reference to the work of autistic scholars. As a result, the arguments are sometimes one-sided and the neurodiversity movement is misrepresented on several fronts. This is a good example of where interdisciplinary engagement between those with in-depth knowledge of ABA and scholars with Critical Autism Studies and Neurodiversity Studies expertise might have better come together to share such expertise rather than risk continued, potentially antagonistic misrepresentation.

However, in keeping with the ambitions of this article we have chosen not to enter into any tit-for-tat here. We are, though, encouraged by Vollmer and Pendergrass (2025, n.p.), writing that “the individuals complaining about ABA are complaining for a reason.” And although perspectives on "quality control" may differ, there are important calls for acknowledgement of poor practice (Nicolosi & Dillenburger, 2025) and the associated need for improved regulation as well as the highlighting of risks to quality of further monetization of autism support via involvement of private equity firms (Morris, 2025).

Although we appreciate the willingness of the editors to engage with the many challenges to ABA, and see their inclusion of this article as demonstrating a readiness to recognize and engage with potentially uncomfortable criticisms, the lack of autistic voices in this journal's special issue is problematic given that it collated “invited essays from leading experts and stakeholders.” It also, perhaps, echoes another, wider problem observed in responses to calls to reform ABA. This is the risk of the ABA industry recognizing the need to go some way towards addressing the many criticisms levied at it but doing so in ways that resemble what Neumeier (2018) has referred to as “neurodiversity-lite” where form takes precedence over substance leading to business as usual rather than meaningful and operationalizable change. The risk, in other words, is that practitioners lean into neurodiversity paradigm language to set themselves apart from critiqued practice without implementing any substantive changes.

To achieve meaningful change, we feel strongly that ABA, often positioned as an agent of normalization (whether intentionally or not), needs to account for the harm this positioning has caused and to listen to the expertise of those it has traditionally treated as subjects, not collaborators, echoing similar calls from other disciplines, such as clinical psychology (Flower et al., 2025). We feel that behavioral theoreticians and practitioners need to reimagine the discipline and to study and borrow from other areas of psychology, from other disciplines that have engaged in critical self-reflection. It is likely that those who are trying to move, in good faith, towards meaningful collaboration with shared aims of a profoundly evolved, just, and compassionate field of behavior analysis require a shared theoretical home. The need is there—and the time is right—for the development of a field that could learn from critical autism, ADHD, and Neurodiversity Studies (CAS, CADS, NDS, respectively), as well as disability studies, in fostering interdisciplinary inquiry, historical self-examination, and an ambition to be transformed for the better. The methodological tools for achieving these aims could include dialogue, historical analysis, critique, and—perhaps above all—the meaningful participation of autistic people as co-constructors of this critical field.

Through interdisciplinary enquiry, ABA would open itself up to wider dialogue and the need for comprehensive learning, and sharing of best practice, in relation to autistic people accessing ABA-based interventions. Recognizing how other fields, such as psychology, medicine, and psychiatry have begun to take notice and respond to the neurodiversity paradigm, ABA should broaden its conversations with the wider field of autism studies, including CAS. There is also the fuller commitment to understanding autism, and autistic people, within a much wider context than simply a reduction to behavior. Behavior analysis would, of course, still focus on the study of behavior, but do so in a way that acknowledges the person as a whole human being living within much broader cultural and sociopolitical contingencies.

Through historical self-examination, ABA could not only cement and develop its social justice origins (Holland, 1978) but also bridge the divide between theory and practice. Further, this would highlight how ABA practice for autism has in our opinion deviated significantly from the intentions of its behaviorist origins, origins that emphasized noncoercion (Skinner, 1948) and a rejection of the medical model (Follette et al., 1992), and become deeply entwined with normalization (Veneziano & Shea, 2023), processes that have been fueled by a tightly gripped virtual monopoly on autism support in certain countries.

Finally, through critique, ABA could engage in meaningful and comprehensive ways with current criticisms and reevaluate unquestioned assumptions. Key in this is a move to view autistic people, and their families, not as subjects of ABA-based practice, but as integral collaborators (Fawcett, 1991). To rise and speak to criticism and tackle head on how ABA can evolve to better serve autistic people rather than simply dismiss voices of concern. This may include, for example, funding research into the proposed link between ABA and harm, and greater appreciation of the key concerns that have an impact on autistic lived experience (Cage et al., 2024). Furthermore, through recognizing the marginalized status of autistic people in ableist social spaces, there would be a renewed commitment to changing environments for autistic people and understanding that greater access and inclusion for autistic people requires bidirectional work on communication, behavior, and societal expectations. Critique could follow the Waltz (2014, p. 1337) working definition of CAS that “The ‘criticality’ comes from investigating power dynamics that operate in discourses around autism, questioning deficit-based definitions of autism, and being willing to consider the ways in which biology and culture intersect to produce ‘disability.’”

Dialogue between CAS and ABA scholars is not easy, but it is possible, and it is necessary. The recent publication of conversation between behavior analysts and critical autism scholars offers a model for what respectful, intellectually rigorous engagement might look like (Suckle et al., 2025). In that dialogue, participants did *not* view consensus as necessary for success. Rather they entered the dialogue with a commitment to simply listen to each other and were pleasantly surprised to find common ground in terms of their commitment to bettering both ABA and the lived experience of autistic people. Other publications going in similar directions indicate that there is a certain momentum building (Bambara et al., 2025; Johnson, 2025).

Let us for now call such a field *Critical Behavioral Studies* (CRIBS). CRIBS would build on existing momentum by creating a formalized space for exchanges. It would be a field in which behavior analysts willing to reflect critically on their practice can engage with autistic scholars on equal footing. It would prioritize social validity as defined by autistic people themselves—in other words, autistic social validity—and reject models of success based on masking or compliance. It would encourage historical accountability, interdisciplinary collaboration, and a rethinking of what behavior analysis might look like when its primary allegiance is to dignity, justice, and the lived realities of the populations served, in this case autistic people.

One potential objection to the current proposal is that behavior analysis is a general science of behavior, not a science of autism. That is of course true but it cannot be denied that greater than 80% of the practitioners of the applied branch of the science work with autistic individuals (Behavior Analyst Certification Board, n.d.). The critical need both for centering autistic perspectives and insisting on autism-specific training in the applied science and practice of behavior analysis is thus essential. The acknowledged risks of insufficient training quality in a field simultaneously experiencing an “influx” of private equity funds, whereby “all decisions regarding the company they control are made in the best interest of their investors, not necessarily the clients served” (Morris, 2025) are hard to overstate. The 40-hr registered behavior technician training—with no autism content for practitioners working with autistic people—would be considered woefully inadequate in many far less sensitive areas: in a field that prides itself on scientific underpinnings, and serves people who, for example, may be autistic, or have intellectual disabilities, or both, it is glaring. When combined with a lack of population-specific knowledge, the risks of ineffective practice, however unintentional, are clear. Given prevailing conflict on arriving at a universal consensus on autism, referred to by Chown et al. (2023) as the autism worldview dilemma, it is also imperative to ensure that such training is underpinned by engagement with neurodiversity scholars, explores autistic ways of being in the world, acknowledges hegemonic norms which may be to the detriment of autistic people, and evidences studies focused on autistic-led priorities for improving autistic people's life experiences.

It is worth noting that the basic tenets we propose for the aspirational field of CRIBS, that is, to center the voices of those being served, to address power dynamics head-on, to use criticism as an opportunity for self-reflection and change, and to center social justice throughout, would presumably be meaningful for any other population. Embracing these principles could bring behavior analysis closer to what the fourth author refers to as the early ambitions of noncoercion, social validity, and social justice as proposed by founders such as B. F. Skinner, Murray Sidman, and Montrose Wolf—which, as Vollmer and Pendergrass (2025) implicitly acknowledge, has not always been sufficiently respected or applied from the perspective of client populations (Chown & Murphy, 2022).

It is no longer feasible either to preserve behavioral interventions as they are, nor to throw the baby out with the bathwater: what remains is to reimagine the field. That reimagining requires creating the intellectual and professional conditions for CRIBS to emerge. This project needs to be moved forward by a new generation of neurodiversity-affirming behavior analysts in collaboration with a broad range of advocates, including autistic behavior analysts and their allies and those who are the most vocal in their criticisms. All need to be willing and able to have the uncomfortable conversations, to learn from mistakes of the past, and to meaningfully and explicitly position behavioral theory and practice as a self-reflective discipline in constant evolution.

## Data Availability

No datasets were generated or analysed during the current study.

## Declarations

### Protection of Human Research Participants

This article does not describe research with human participants.
